# 单孔与三孔胸腔镜肺癌根治术对患者术后疼痛及短期生活质量的对比研究

**DOI:** 10.3779/j.issn.1009-3419.2016.03.02

**Published:** 2016-03-20

**Authors:** 志鹏 郝, 奕欣 蔡, 圣灵 付, 霓 张, 向宁 付

**Affiliations:** 430030 武汉，华中科技大学同济医学院附属同济医院胸外科 Department of Thoracic Surgery, Tongji Hospital, Tongji Medical College, Huazhong University of Science and Technology, Wuhan 430030, China

**Keywords:** 肺肿瘤, 胸腔镜手术, 术后疼痛, 生活质量, Lung neoplasms, Video-assisted thoracic surgery, Post-operative pain, Quality of life

## Abstract

**背景与目的:**

近年来在传统三孔胸腔镜的基础上，单孔胸腔镜术式发展迅速并渐用于肺癌根治性切除，其与传统胸腔镜术式相比的临床应用优势也为关注的热点。本研究针对单孔胸腔镜肺癌根治术对患者术后疼痛及短期生活质量的影响进行初步探讨。

**方法:**

选取2015年3月-2015年9月在我科同诊疗组连续行单孔胸腔镜（单孔组）或三孔胸腔镜（三孔组）肺癌根治术的非小细胞肺癌患者216例，其中单孔组115例，三孔组101例。对比两组的临床及手术资料，以视觉模拟评分（visual analogue scale, VAS）法评估两组患者术后第3天、第7天时疼痛的最小（VASmin-d3、d7）及最大（VASmax-d3、d7）值，肺癌治疗功能性量表（Functional Assessment of Cancer Treatment-Lung, FACT-L）中文版v4.0评测两组患者术前及术后三月的生活质量，对比两组术后三月切口麻木发生率及患者对切口外观的满意度。

**结果:**

两组患者的一般临床资料无差异，均无围手术期死亡病例，单孔组手术时间（157.62±19.50）min较三孔组（116.00±17.32）min更长（*P*＜0.001），但术后胸管置管时间和术后住院时间在单孔组[(4.37±1.65) d, (9.87±1.25) d]均明显短于三孔组[(5.54±1.57) d, (10.43±1.43) d]（*P*=0.020, *P*=0.004）；两组患者术后VASmin-d3无显著差异，但单孔组VASmin-d7及VASmax-d3、d7[(1.46±0.29), (3.75±0.54), (2.43±0.53)]均低于三孔组[(1.58±0.30), (3.93±0.51), (2.62±0.62); *P*=0.003; *P*=0.011; *P*=0.018]。FACT-L评分显示术后三月单孔组患者功能状态、情感状态和整体生活质量得分[(20.94±2.22), (19.88±1.70), (108.09±4.58)]均高于三孔组患者[(20.24±1.92), (19.36±1.67), (106.88±4.17); *P*=0.014; *P*=0.024; *P*=0.045]，而生理状态、社会/家庭状态及肺癌相关症状评分两组并无差异。与三孔组比较，单孔组术后三月切口麻木发生率（24.3% *vs* 38.6%）更低（*P*=0.024），患者对切口的满意度更高（78.3% *vs* 65.3%, *P*=0.035）。

**结论:**

与三孔胸腔镜相比，单孔胸腔镜肺癌根治术能够减轻患者术后疼痛，改善术后短期生活质量，在肺癌的外科治疗中有一定临床应用价值。

## Abstract (en)

**Background and objective:**

In the recent years, uniportal video-assisted thoracic surgery (uniportal-VATS) is developing rapidly on the basis of traditional three portal VATS and has been applied for radical resection of lung cancer, its superiority on the clinical application compared with traditional VATS is also become the focus of the attention, the aim of this study is to preliminary investigate the effect of uniportal -VATS on post-operative pain and short-term quality of life.

**Methods:**

Data of 216 patients who underwent uniportal-VATS (*n*=115) or three portal VATS (3P-VATS)(*n*=101) for radical resection of non-small cell lung cancer (NSCLC) were analyzed. The clinical and operative data were assessed, visual analogue scale (VAS) was used to evaluate the minimum and maximum pain score (VASmin and VASmax) on the 3^rd^ and 7^th^ post-operative day. Chinese version 4.0 of Functional Assessment of Cancer Treatment-Lung (FACT-L) was applied to evaluate the short-term quality of life preoperatively and 3 months after operation, respectively. Incidence of incision numbness and satisfaction rate of incision appearance were compared between the two groups 3 months after operation.

**Results:**

Both groups were similar in clinical characteristics, there were no perioperative death in two groups. While the operative time of uniportal-VATS group (157.62±19.50) min was longer than that of the 3P-VATS group [(116.00±17.32) min, *P* < 0.001], the chest tube duration and postoperative hospital stay of uniportal-VATS group [(4.37±1.65) d, (9.87±1.25) d] were both shorter than those of 3P-VATS group [(5.54±1.57) d, (10.43±1.43) d, *P*=0.020, *P*=0.004]. No statistically significant difference was reported in VASmin-d3 [(1.98±0.57) *vs* (2.09±0.59), *P*=0.148] between the two groups, however, VASmin-d7 and VASmax-d3, d7 were all significantly less in uniportal-VATS group [(1.46±0.29), (3.75±0.54), (2.43±0.53)] than in 3P-VATS group [(1.58±0.30), (3.93±0.51), (2.62±0.62), *P*=0.003, *P*=0.011, *P*=0.018]. FACT-L assessment indicated that the scores of patients' functional status, emotional status and overall quality of life 3 months after the surgery in uniportal VATS group [(20.94±2.22), (19.88±1.70), (108.09±4.58)] were all higher than those in 3P-VATS group [(20.24±1.92), (19.36±1.67), (106.88±4.17), *P*=0.014, *P*=0.024, *P*=0.045], there were no significant differences between the two groups in physical status, social and family conditions as well as in lung cancer subscale. Compared with 3P-VATS group, the incidence of incision numbness was lower (24.3% *vs* 38.6%, *P*=0.024) and satisfaction rate of incision was higher (78.3% *vs* 65.3%, *P*=0.035) in uniportal-VATS group at the 3 months follow-up.

**Conclusion:**

Compared with 3P-VATS, uniportal-VATS radical lobectomy could reduce post-operative pain and improve short-term postoperative quality of life, it has practical clinical value in surgical therapy of lung cancer.

单孔胸腔镜手术（uniportal video-assisted thoracoscopic surgery, uniportal-VATS）近年来发展迅速，手术适用范围渐扩大至肺叶切除、肺癌根治性切除等较复杂术式^[1-3]^。与传统三孔胸腔镜（three portal video-assisted thoracoscopic surgery, 3P-VATS）相比，Uniportal-VATS有其自身的特点及优势^[4]^，切口的减少可能改善患者术后疼痛，而对肺癌患者除需确保手术的安全性和肿瘤切除的彻底性外，追求更高的术后生活质量同样为治疗目标之一。我们在本研究中对比分析Uniportal-VATS和3P-VATS肺癌根治术对患者术后疼痛及短期生活质量的影响，进一步探讨Uniportal-VATS在肺癌患者外科治疗中的应用价值。

## 1 资料与方法

### 1.1 对象

分析2015年3月-2015年9月在华中科技大学同济医学院附属同济医院胸外科同一手术组连续行肺叶切除+系统性淋巴结清扫的肺癌患者资料289例。入选标准：①术后病理诊断确诊为非小细胞肺癌；②所有患者均行单孔胸腔镜或三孔胸腔镜肺叶切除（或全肺切除）+系统性淋巴结清扫术。排除标准：①术中中转开胸患者；②术后因出血等并发症再次手术患者；③有肿瘤转移患者。患者术后病理分期按国际肺癌研究协会（International Association for the Study of Lung Cancer, IASLC）2009年第七版分期标准。手术方式由术者根据患者病情决定。术后3个月随访时有3例患者失访，最终纳入216例患者资料进行分析，其中单孔胸腔镜组（单孔组）115例，三孔胸腔镜组（三孔组）101例。患者一般临床资料见表 1。

**1:** 两组患者临床特征 The characteristics of the two groups

Characteristics	Uniportal-VATS (*n*=115)	3P-VATS (*n*=101)	*P*
Age (yr)	60.1±5.7	61.0±5.2	0.230
Gender			0.585
Male	68 (59.1%)	56 (55.4%)	
Female	47 (40.9%)	45 (44.6%)	
Smoking	61 (53.0%)	52 (51.5%)	0.819
BMI（kg/m^2^）	21.76±1.65	22.02±1.47	0.205
Tumor location			
RUL	36 (31.3%)	26 (25.7%)	0.367
RML	10 (8.7%)	8 (7.9%)	0.837
RLL	19 (16.5%)	19 (18.8%)	0.659
LUL	29 (25.2%)	23 (22.8%)	0.675
LLL	21 (18.3%)	25 (24.8%)	0.245
Concomitant diseases			
Coronary disease	8 (7.0%)	7 (6.9%)	0.994
Hypertension	29 (25.2%)	22 (21.8%)	0.553
COPD	60 (52.2%)	46 (45.5%)	0.331
Diabetes	8 (7.0%)	5 (5.0%)	0.536
Histology			
Squamousl carcinoma	51 (44.3%)	42 (41.6%)	0.682
Adenocarcinoma	56 (48.7%)	53 (52.5%)	0.579
Other type	8 (6.9%)	6 (5.9%)	0.762
TNM stage			
Ⅰ	79 (68.7%)	66 (65.3%)	0.601
Ⅱ	15 (13.0%)	13 (12.9%)	0.970
Ⅲ	21 (18.3%)	22 (21.8%)	0.518
RUL: right upper lobe; RML: right middle lobe; RLL: right lower lobe; LUL: left upper lobe; LLL: left lower lobe; BMI: body mass index; COPD: chronic obstructive pulmonary disease.			

### 1.2 方法

#### 1.2.1 手术方法（图 1）

**1:**
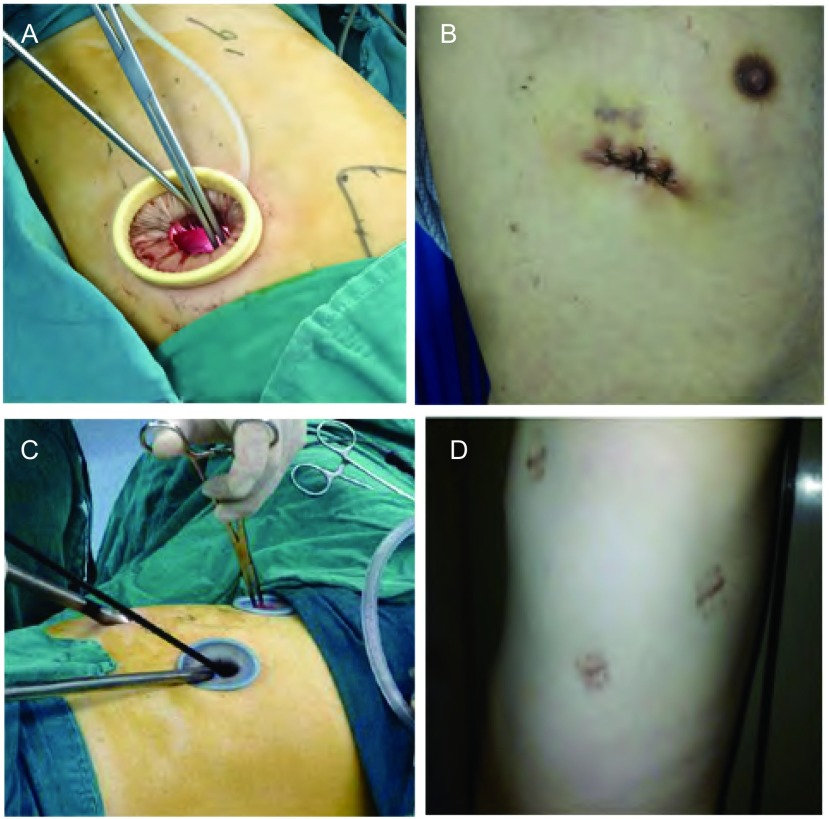
单孔与三孔胸腔镜术中及术后情况。A：单孔胸腔镜术中操作；B：单孔胸腔镜术后伤口；C：三孔胸腔镜术中操作；D：三孔胸腔镜术后伤口。 Procedure and incision of Uniportal-VATS and 3P-VATS. A: procedure of Uniportal-VATS; B: incision of Uniportal-VATS after operation; C: procedure of 3P-VATS; D: incision of 3P-VATS after operation.

患者均侧卧位，双腔气管插管全身麻醉。①单孔组（Uniportal-VATS）中，肺上叶及中叶手术的切口位于术侧第5肋间腋前线与腋中线之间，下叶肺部手术取术侧第4肋间，切口长约3 cm，切开皮肤后经肋间肌中部切断进入胸腔，切口置软质切口保护套，完全在腔镜下完成肺叶切除及系统性淋巴结清扫，术毕经切口留置胸管引流。②三孔组（3P-VATS）取腋中线第7肋间1.5 cm切口为观察孔，肩胛线第7或第8肋间1.0 cm切口及腋前线第3肋间1.5 cm切口为操作孔完成手术，术毕经观察孔及操作孔各置胸管一根引流。按照中国原发性肺癌诊疗规范（2015版）^[5]^的要求行系统性淋巴结清扫，除N1站淋巴结外，N2站淋巴结左胸清扫范围为4L、5-9组，右胸手术清扫范围为2R、3a、3p、4R、7-9组。所有患者术后使用舒芬太尼自控静脉镇痛泵。

#### 1.2.2 术后处理

术后按胸外科常规处理，鼓励患者早期咳嗽及下床活动，术后48 h停用自控静脉镇痛泵。胸片提示双肺复张良好且胸管引流量≤150 mL时拔除胸管。TNM分期为高危Ⅰb期及Ⅱ期-Ⅲ期的NSCLC肺癌患者建议其术后3周-4周于肿瘤科行辅助化疗。

#### 1.2.3 观察指标

患者临床特征包括性别、年龄、身体质量指数（body mass index, BMI）、吸烟史、术前合并疾病[包括冠心病、高血压、慢性阻塞性肺疾病（chronic obstructive pulmonary disease, COPD）、糖尿病]、肿瘤部位、肿瘤组织学类型和TNM分期。统计两组手术方式、手术时间、术中出血量、淋巴结清扫数、胸管留置时间、术后并发症情况（包括肺部感染、肺漏气＞5 d、肺不张、心律失常、皮下气肿）、术后住院时间。

术后疼痛评估采取视觉模拟评分法（visual analogue scale, VAS），使用VAS游动标尺，两端分别为0分和10分，分别代表无痛和难以忍受的最剧烈疼痛，标尺背面对应一条由绿色向红色渐变的彩色直线反映患者疼痛的程度。评测时由患者根据自己的疼痛感受在直线上指出相应位置，反面对应数值即为疼痛评分值。记录患者术后第3天、第7天疼痛的最小值（minimum of VAS, VASmin）及最大值（maximum of VAS, VASmax）。使用肺癌治疗功能性量表（Functional Assessment of Cancer Treatment-Lung, FACT-L）中文版v4.0评估患者生活质量，FACT-L问卷量表由五个部分组成，分别代表患者的生理状态、社会/家庭状态、功能状态、情感状态及肺癌相关症状五个方面内容，每个部分由6个-9个简单条目组成，各条目采取五级评定法：一点也不、有一点、有些、相当、非常，患者参照自身情况对每个条目勾选完毕后进行评分，积极的正向条目分别代表 0分-4分，不良的负面条目分别代表 4分-0分，得分越高提示生活质量越好。术前及术后三月随访时由医护人员指导患者完成量表，同时在术后3个月随访患者切口麻木情况及对手术切口满意度。

### 1.3 统计学方法

统计数据采用SPSS 19.0统计软件分析，计量资料以均数±标准差（mean±SD）表示，组间比较采用独立样本*t*检验；计数资料以实际例数及所占百分比表示，两组率比较采用χ^2^检验，以*P*＜0.05为差异有统计学意义。

## 2 结果

### 2.1 两组患者手术临床资料分析

两组均无围手术期死亡患者。单孔组手术时间[（157.62±19.50）min]较三孔组[（116.00±17.32）min]更长（*P*＜0.001），但两组患者手术方式、术中出血量、清扫淋巴结数及术后并发症发生率无统计学差异。单孔组术后胸管置管时间和住院时间[(4.37±1.65) d, (9.87±1.25) d]均较三孔组[(5.54±1.57) d, (10.43±1.43) d]明显缩短（*P*=0.020, *P*=0.004）（表 2）。

**2:** 两组患者手术及术后临床资料 Comparison of operative and post-operative clinical data between uniportal-VATS and 3P-VATS group

Item	Uniportal-VATS (*n*=115)	3P-VATS (*n*=101)	*P*
Operation time (min)	157.62±19.50	116.00±17.32	< 0.001
Blood loss (mL)	77.74±64.26	81.15±46.76	0.660
Operation method			
Single lobectomy	108 (93.9%)	92 (91.1%)	0.429
Bilobectomy	6 (5.2%)	7 (6.9%)	0.597
pneumonectomy	1 (0.9%)	2 (2.0%)	0.910
Number of lymph nodes	19.65±2.84	20.33±2.99	0.091
Chest tube duration (d)	4.37±1.65	5.54±1.57	0.020
Postoperative hospital stay (d)	9.87±1.25	10.43±1.43	0.004
Complication			
Lung infection	7 (6.1%)	8 (7.9%)	0.597
Prolonged air leaking > 5 d	3 (2.6%)	5 (5.0%)	0.584
Atelectasis	2 (1.7%)	3 (3.0%)	0.883
Arhythmia	14 (12.2%)	16 (15.8%)	0.437
Subcutaneous emphysema	4 (3.5%)	4 (4.0%)	0.862

### 2.2 两组患者术后早期疼痛情况评估

两组术后VAS疼痛评分详见表 3。VASmin评测方面，术后第3天疼痛最小值（VASmin-d3）两组无统计学差异，但单孔组VASmin-d7（1.46±0.29）低于三孔组[(1.58±0.30), *P*=0.003]。单孔组VASmax-d3、d7[(3.75±0.54), (2.43±0.53)]均低于三孔组[(3.93±0.51), (2.62±0.62)]，差异具有统计学意义（*P*=0.011, *P*=0.018）。

**3:** 两组患者术后疼痛分析 Analysis of post-operative pain between Uniportal-VATS and 3P-VATS group

	Uniportal-VATS (*n*=115)	3P-VATS (*n*=101)	*P*
VAS_min_-d3	1.98±0.57	2.09±0.59	0.148
VAS_max_-d3	3.75±0.54	3.93±0.51	0.011
VAS_min_-d7	1.46±0.29	1.58±0.30	0.003
VAS_max_-d7	2.43±0.53	2.62±0.62	0.018

### 2.3 两组患者术前及术后3个月生活质量分析

所有患者FACT-L评分详见表 4。两组患者术前生活质量基线水平相似。术后3个月随访，单孔组患者在功能和情感状态[(20.94±2.22), (19.88±1.70)]评分上优于三孔组[(20.24±1.92), (19.36±1.67)]，差异有统计学意义（*P*=0.014, *P*=0.024），而生理状态、社会/家庭及肺癌相关症状评分两组无差异，对生活质量的整体评价单孔组（108.09±4.58）优于三孔组（106.88±4.17）（*P*=0.045）。

**4:** 两组患者手术前后生活质量对比 Comparison of patients'preoperative and postoperative quality of life between Uniportal-VATS and 3P-VATS group

Item	Pre-operation				Three months post-operation		
	Uniportal-VATS (*n*=115)	3P-VATS (*n*=101)	*P*		Uniportal-VATS *n*=115)	3P-VATS (*n*=101)	*P*
Physical status	19.27±1.90	19.48±1.94	0.432		20.69±1.92	20.28±1.80	0.108
Social and family conditions	18.83±1.90	18.65±1.74	0.489		22.77±1.98	22.88±1.02	0.583
Emotion status	13.50±1.31	13.71±1.40	0.259		19.88±1.70	19.36±1.67	0.024
Functional status	14.40±1.46	14.47±1.40	0.739		20.94±2.22	20.24±1.92	0.014
Lung cancer subscale	23.19±1.58	23.00±1.72	0.395		23.82±1.06	24.13±1.68	0.111
Overall score	89.19±4.52	89.31±2.73	0.839		108.09±4.58	106.88±4.17	0.045
Incision numbness	-	-	-		28 (24.3%)	39 (38.6%)	0.024
Satisfaction with incision	-	-	-		90 (78.3%)	66 (65.3%)	0.035

术后3个月单孔组切口麻木发生率（24.3%）明显低于三孔组（38.6%）（*P*=0.024），而该组患者对切口满意度（78.3%）则显著高于三孔组（65.3%）（*P*=0.035）（表 4）。

## 3 讨论

肺癌在全球和我国的发病率及癌症致死率均居首位^[6, 7]^，解剖性肺叶切除+系统性淋巴结清扫是目前临床治愈肺癌的重要方法，胸腔镜手术的微创优势使其在肺癌外科中占据重要地位。Uniportal-VATS自2004年Rocco^[8]^首次报道以来发展迅速，从最初的肺肿块楔形切除扩大应用至肺癌根治术、支气管或肺动脉成形术等复杂手术^[9]^。胸腔镜技术的发展需在保证手术安全性和疗效的基础上展现其“微”创伤优势，不仅体现在切口数量的减少和长度的缩短，还应能减轻患者术后不适、促进快速康复，NSCLC的外科治疗不仅需通过手术达到肿瘤根治性切除的目的，追求更高的术后生活质量同样重要。

肺癌根治性切除需完成解剖性肺叶切除及系统性淋巴结清扫。本研究中单孔组在手术方式及淋巴结清扫个数方面和三孔组相似，两组均无围手术期死亡病例，且在术中出血量及术后并发症发生率上两组间亦无明显差异，提示Uniportal-VATS和传统3P-VATS一样能够胜任肺癌根治术且未增加手术的风险。

由传统三孔操作转变至单孔操作，因器械投射至胸腔内的操作平面较小，且所有器械经由一个孔道进出易相互干扰，其操作性和传统三孔腔镜相比存在一定的差异，手术者需经历一定的适应和调整期，但具有多孔胸腔镜操作经验手术者经较短学习周期即可掌握^[10, 11]^。本研究为连续入组的患者，单孔组中未排除开展单孔手术之初的手术人群，由于前期对单孔操作流程和器械应用的摸索，这些患者的手术时间较后期患者会更长，可能是导致单孔组手术时间较三孔组更长的原因，但两组手术时间相差尚在1 h以内，术中出血量无差异，亦未发现单孔组患者术后并发症发生率更高，其手术的安全性能够得到保证，不会明显增加患者手术费用。有研究显示单孔和三孔胸腔镜的手术时间相比并无差异^[10]^，相信随着单孔胸腔镜技术的进步和器械的改进，其手术时间能得到进一步缩短。

术后疼痛是困扰胸外科医生和患者的常见问题，有研究显示Uniportal-VATS治疗气胸、纵隔肿块或行肺肿块楔形切除，术后疼痛较3P-VATS更轻^[12, 13]^。胸外科患者术后疼痛常在咳嗽时达峰值，而卧床休息时疼痛有所缓解。本研究将患者对疼痛的主观感受细化为最小及最大值分别比较，更详细分析Uniportal-VATS对术后疼痛的影响，结果发现术后单孔组VASmax均明显低于三孔手术组，提示Uniportal-VATS更能减轻患者术后早期咳嗽时的痛觉。术后疼痛会影响患者咳嗽排痰及早期活动，阻碍术后快速康复，肋间神经损伤是导致疼痛的主要原因之一^[14]^，我们分析可能因单孔胸腔镜手术仅切开一个肋间，降低了肋间神经受损的概率；肋间肌由中部切断而未紧贴下一肋上缘，降低了手术器械对肋骨骨膜的损伤；软质切口保护套的使用避免了硬质Troca对骨膜及肋间神经的卡压，这些都有助于减轻患者术后疼痛，增强其有效咳嗽能力，促进肺复张而尽早消除胸腔内残腔，这也可能是单孔组术后胸管置管时间更短的原因；单孔组VASmin-7亦低于三孔组，使患者能更快摆脱疼痛的困扰。这些因素的叠加缓解了患者因疼痛产生的不良生理和心理应激反应，使其能尽快建立康复信心，缩短术后住院时间。

和传统3P-VATS相比，Uniportal-VATS还可改善术后短期生活质量。我们所选的FACT-L量表由美国芝加哥Rush-Presbyterian-St. Luke医学中心研制^[15]^，是目前应用较多的评价肺癌患者生活质量的量表之一^[16]^。本研究中两组患者术前生活质量基线水平相似，术后3个月单孔组患者的功能和情感状态较三孔组更佳，整体生活质量更优于三孔胸腔镜。可能因：①3 cm的手术切口及术后更轻的疼痛使患者更容易接受单孔胸腔镜术式，从主观意愿上认为该术式对身体创伤较小，单孔组患者对切口更高的满意度也从侧面反映了患者对该术式的认可程度，使患者术后更容易恢复积极向上的正面情绪，更快恢复至正常的生活和工作状态。②单孔组患者术后切口麻木发生率更低。切口麻木是胸外科术后常见不良反应，对患者术后生活造成不同程度的影响，Uniportal-VATS可能通过减少肋间神经受损，而降低切口麻木发生率，使患者更易摆脱术后机体不适及其带来的不良心理情绪，有助于改善术后生活质量。

本研究发现单孔胸腔镜肺癌根治术能达到传统三孔胸腔镜相同的手术切除效果，且能保证手术安全性，在改善患者术后疼痛及近期生活质量方面更优于传统三孔胸腔镜。已有研究发现早期肺癌患者行Uniportal-VATS治疗，术后30个月的生存率为90%^[17]^，术后2年生存率和传统胸腔镜手术相当^[18]^，在此基础上我们尚需进一步观察Uniportal-VATS对肺癌患者远期生活质量及长期生存率的影响，探索和评估其在肺癌外科治疗的临床应用前景。
